# Adverse outcomes following pelvic fracture: the critical role of frailty

**DOI:** 10.1007/s00068-023-02355-0

**Published:** 2023-08-29

**Authors:** Maximilian Peter Forssten, Babak Sarani, Ahmad Mohammad Ismail, Yang Cao, Marcelo A. F. Ribeiro, Frank Hildebrand, Shahin Mohseni

**Affiliations:** 1 School of Medical Sciences, Orebro University, 701 82 Orebro, Sweden; 2 Department of Orthopedic Surgery, Orebro University Hospital, 701 85 Orebro, Sweden; 3 Center of Trauma and Critical Care, George Washington University, Washington, DC USA; 4 Clinical Epidemiology and Biostatistics, School of Medical Sciences, Faculty of Medicine and Health, Orebro University, 701 82 Orebro, Sweden; 5 Khalifa University and Gulf Medical University, Abu Dhabi, United Arab Emirates; 6 Department of Orthopedics, Trauma and Reconstructive Surgery, University Hospital RWTH Aachen, Pauwelsstrasse 30, 52074 Aachen, Germany; 7 Division of Trauma, Critical Care and Acute Care Surgery, Department of Surgery, Sheikh Shakhbout Medical City, Mayo Clinic, Abu Dhabi, United Arab Emirates

**Keywords:** Pelvic fracture, Frailty, Geriatric patients, Mortality, Morbidity, Failure-to-rescue

## Abstract

**Purpose:**

Pelvic fractures among older adults are associated with an increased risk of adverse outcomes, with frailty likely being a contributing factor. The current study endeavors to describe the association between frailty, measured using the Orthopedic Frailty Score (OFS), and adverse outcomes in geriatric pelvic fracture patients.

**Methods:**

All geriatric (65 years or older) patients registered in the 2013–2019 Trauma Quality Improvement Program database with an isolated pelvic fracture following blunt trauma were considered for inclusion. An isolated pelvic fracture was defined as any fracture in the pelvis with a lower extremity AIS ≥ 2, any abdomen AIS, and an AIS ≤ 1 in all other regions. Poisson regression models were employed to determine the association between the OFS and adverse outcomes.

**Results:**

A total of 66,404 patients were included for further analysis. 52% (*N* = 34,292) were classified as non-frail (OFS 0), 32% (*N* = 21,467) were pre-frail (OFS 1), and 16% (*N* = 10,645) were classified as frail (OFS ≥ 2). Compared to non-frail patients, frail patients exhibited a 88% increased risk of in-hospital mortality [adjusted IRR (95% CI): 1.88 (1.54–2.30), *p* < 0.001], a 25% increased risk of complications [adjusted IRR (95% CI): 1.25 (1.10–1.42), *p* < 0.001], a 56% increased risk of failure-to-rescue [adjusted IRR (95% CI): 1.56 (1.14–2.14), *p* = 0.006], and a 10% increased risk of ICU admission [adjusted IRR (95% CI): 1.10 (1.02–1.18), *p* = 0.014].

**Conclusion:**

Frail pelvic fracture patients suffer from a disproportionately increased risk of mortality, complications, failure-to-rescue, and ICU admission. Additional measures are required to mitigate adverse events in this vulnerable patient population.

**Supplementary Information:**

The online version contains supplementary material available at 10.1007/s00068-023-02355-0.

## Introduction

A demographic shift is currently underway worldwide, with a growing proportion of geriatric individuals in the population. This trend can be attributed to various factors, such as advancements in medicine and improved living conditions. In developed countries, the geriatric population is growing at a faster rate than the general population and is expected to continue to do so [1–3]. This demographic shift is also evident in the incidence of pelvic fractures, which has been steadily increasing, especially among older individuals [4–6]. This is particularly worrying considering the elevated risk of adverse outcomes after traumatic insults and subsequent surgical interventions in this patient population [7]. The mortality rate following low energy pelvic ring fractures can be as high as 27%, comparable to that following hip fracture in geriatric patients [5–13]. Despite the significant disease burden associated with pelvic fractures, they have not been as thoroughly studied in the geriatric population [5, 14].

Geriatric pelvic fracture patients present a heterogeneous patient population with varying prognoses following injury based on fracture pattern and preexisting comorbidities [5, 6, 8–12]. One potential avenue for guiding patient care is stratification of patients based on their intrinsic risk of adverse outcomes. Frailty, a condition characterized by a reduced physiological reserve to withstand external stressors [15–18], has previously been found to be relatively common in the geriatric trauma patients. It has also been associated with an increased risk of both morbidity and mortality [8, 19–21]. The aim of the current investigation was therefore to determine the association between frailty, measured using the Orthopedic Frailty Score (OFS), and adverse outcomes in geriatric pelvic fracture patients. We hypothesize that an increasing OFS is associated with increasing risk of morbidity and mortality in geriatric patients who have sustained a pelvic fracture.

## Methods

The need for ethical approval for the current analysis was waived by the relevant ethical review board due to the retrospective, anonymized nature of the utilized dataset. All aspects of the current investigation adhered to the Declaration of Helsinki and STrengthening the Reporting of OBservational studies in Epidemiology guidelines [22]. Data were obtained from the American College of Surgeons’ 2013–2019 Trauma Quality Improvement Program (TQIP) database. This dataset allowed for the retrieval of variables pertaining to patient demographics, clinical characteristics, injury severity measured using the Abbreviated Injury Severity Score (AIS), interventions, and outcomes. All geriatric (65 years or older) patients who suffered an isolated pelvic fracture subsequent to a blunt trauma were eligible for inclusion. An isolated pelvic fracture was defined as any fracture in the ilium, ischium, pubis, sacrum, coccyx, or acetabulum with a lower extremity AIS ≥ 2, any abdomen AIS, and an AIS ≤ 1 in all other regions. Patients with any abdomen AIS were included in order to avoid excluding patients with intraabdominal vascular injuries resulting from the pelvic fracture. Patients were excluded if they had an AIS of 6 in the lower extremity or abdomen, as these injuries are generally not considered survivable.

### Calculating the Orthopedic Frailty Score

The degree of frailty for each patient was estimated using the Orthopedic Frailty Score. This score makes use of five dichotomous variables: age ≥ 85 years old, institutionalization, non-independent functional status, congestive heart failure, and a history of malignancy (excluding non-invasive neoplasms of the skin). Institutionalization was defined as the admission origin being from an institution such as a nursing home, long-term care facility, or other group living arrangement. Patients received one point for each variable present, resulting in a maximum total score of 5 [23].

### Statistical analysis

Patients were separated into groups based on their OFS: non-frail (OFS = 0), pre-frail (OFS = 1), and frail (OFS ≥ 2) [24]. Continuous variables, being non-normally distributed, were summarized as medians and interquartile ranges (IQRs), while categorical variables were presented as counts and percentages. Statistical analysis was performed using the Kruskal–Wallis test for continuous variables and either the Chi-squared test or Fisher’s exact test for categorical variables, as appropriate. The primary outcome of interest was in-hospital mortality, and secondary outcomes included in-hospital complications (myocardial infarction, cardiac arrest with CPR, stroke, deep vein thrombosis, pulmonary embolism, acute respiratory distress syndrome, urinary tract infection, pneumonia, surgical site infection, sepsis, decubitus ulcer, unplanned intubation, unplanned admission to the OR, unplanned admission to the ICU), failure-to-rescue (FTR), and ICU admission. FTR was defined as in-hospital mortality following a complication.

To assess the association between frailty and adverse outcomes, Poisson regression models were employed in order to minimize potential confounding. Explanatory variables included frailty (measured using the OFS), age, sex, race, highest AIS in each region, type of fracture, surgical and angiographic procedures, hypertension, previous myocardial infarction, history of peripheral vascular disease, cerebrovascular disease, dementia, chronic obstructive pulmonary disease, smoking status, chronic renal failure, diabetes mellitus, cirrhosis, coagulopathy, drug use disorder, alcohol use disorder, major psychiatric illness, and advanced directives limiting care. The response variable was either in-hospital mortality, complications, FTR, or ICU admission. Results were presented as adjusted incidence rate ratios (IRRs) and corresponding 95% confidence intervals (CIs) calculated using robust standard errors [25]. These analyses were also repeated in several subgroup analyses. The first subgroup consisted of all patients who suffered a pelvic fracture after a ground-level fall (GLF). The second subgroup consisted of patients who were managed conservatively, defined as not undergoing surgical fracture fixation, vascular surgery, any angiographic procedure, or laparotomy/pelvic packing for hemorrhage control. The final subgroup consisted of patients with an abdomen AIS ≤ 1.

A two-sided *p*-value < 0.05 was considered statistically significant. Missing data were managed using multivariate imputation by chained equations, with a total of seven complete datasets being generated, assuming data were missing at random. Analyses were performed using the *tidyverse*, *parallel*, *mice*, and *sandwich* packages in the statistical software R 4.0.5 (R Foundation for Statistical Computing, Vienna, Austria) [26].

## Results

Subsequent to applying the inclusion and exclusion criteria, 66,404 patients were included for further analysis. 52% (*N* = 34,292) were classified as non-frail, 32% (*N* = 21,467) were pre-frail, and 16% (*N* = 10,645) were classified as frail. Median age tended to increase with a higher OFS (pre-frail and frail vs non-frail: 85 [79–87] and 86 [84–88] vs 76 [70–81] years old, *p* < 0.001). Additionally, there was a significant difference in the distribution of sex and race among the three groups, with a higher proportion of females (pre-frail and frail vs. non-frail: 74.6% and 77.3% vs 66.1%, *p* < 0.001) and White patients (pre-frail and frail vs. non-frail: 89.5% and 91.9% vs 88.3%, *p* < 0.001) in the pre-frail and frail groups, compared to the non-frail group. The prevalence of all comorbidities was also highest among frail patients, apart from cirrhosis and substance use disorders (Table 1). Frail patients were generally more severely injured than pre-frail patients (Lower extremity AIS ≥ 3: 16.5% vs 14.5%, *p* < 0.001). Pre-frail and frail patients were also more likely to have suffered a pubis fracture (67.7% and 67.6% vs 62.5%, *p* < 0.001) and less likely to have suffered an acetabulum fracture (26.6% and 26.8% vs 34.7%, *p* < 0.001), compared to non-frail patients. Surgical and angiographic interventions were also significantly less common the higher the degree of frailty (Table 2).

**Table 1:** Demographics of geriatric patients with pelvic fractures

	Non-frail (*N* = 34,292)	Pre-frail (*N* = 21,467)	Frail (*N* = 10,645)	*p* value
Age, median [IQR]	76 [70–81]	85 [79–87]	86 [84–88]	< 0.001
Sex, *n* (%)				< 0.001
Female	22,675 (66.1)	16,005 (74.6)	8224 (77.3)	
Male	11,612 (33.9)	5457 (25.4)	2417 (22.7)	
Missing	5 (0.0)	5 (0.0)	4 (0.0)	
Race, *n* (%)				
White	30,283 (88.3)	19,207 (89.5)	9785 (91.9)	< 0.001
Black	1421 (4.1)	874 (4.1)	361 (3.4)	0.001
Asian	598 (1.7)	354 (1.6)	129 (1.2)	< 0.001
American Indian	160 (0.5)	86 (0.4)	37 (0.3)	0.192
Pacific islander	34 (0.1)	17 (0.1)	6 (0.1)	0.381
Other	1239 (3.6)	612 (2.9)	233 (2.2)	< 0.001
Missing	383 (1.1)	234 (1.1)	56 (0.5)	
Hypertension, *n* (%)	20,265 (59.1)	14,593 (68.0)	7576 (71.2)	< 0.001
Previous myocardial infarction, *n* (%)	517 (1.5)	448 (2.1)	241 (2.3)	< 0.001
Congestive heart failure, *n* (%)	0 (0.0)	2560 (11.9)	3507 (32.9)	< 0.001
History of peripheral vascular disease, *n* (%)	418 (1.2)	387 (1.8)	308 (2.9)	< 0.001
Cerebrovascular disease, *n* (%)	1440 (4.2)	1467 (6.8)	887 (8.3)	< 0.001
Dementia, *n* (%)	2339 (6.8)	3541 (16.5)	3534 (33.2)	< 0.001
Non-independent functional status, *n* (%)	0 (0.0)	4939 (23.0)	6971 (65.5)	< 0.001
Institutionalized, *n* (%)	0 (0.0)	1814 (8.5)	4800 (45.1)	< 0.001
History of malignancy, *n* (%)	0 (0.0)	742 (3.5)	625 (5.9)	< 0.001
Chronic obstructive pulmonary disease, *n* (%)	4199 (12.2)	3331 (15.5)	1904 (17.9)	< 0.001
Current smoker, *n* (%)	3434 (10.0)	1438 (6.7)	426 (4.0)	< 0.001
Chronic renal failure, *n* (%)	1037 (3.0)	1052 (4.9)	604 (5.7)	< 0.001
Diabetes mellitus, *n* (%)	7178 (20.9)	4679 (21.8)	2380 (22.4)	0.002
Cirrhosis, *n* (%)	350 (1.0)	204 (1.0)	102 (1.0)	0.676
Coagulopathy, *n* (%)	2348 (6.8)	2010 (9.4)	1150 (10.8)	< 0.001
Drug use disorder, *n* (%)	335 (1.0)	183 (0.9)	71 (0.7)	0.010
Alcohol use disorder, *n* (%)	988 (2.9)	313 (1.5)	90 (0.8)	< 0.001
Major psychiatric illness, *n* (%)	3209 (9.4)	2442 (11.4)	1592 (15.0)	< 0.001
Advanced directive limiting care, *n* (%)	1322 (3.9)	1992 (9.3)	2107 (19.8)	< 0.001

Non-frail, pre-frail, and frail are defined as OFS 0, OFS 1, and OFS ≥ 2

**Table 2:** Clinical characteristics of geriatric patients with pelvic fractures

	Non-frail (*N* = 34,292)	Pre-frail (*N* = 21,467)	Frail (*N* = 10,645)	*p* value
Head AIS, *n* (%)				< 0.001
Injury not present	32,043 (93.4)	20,035 (93.3)	9828 (92.3)	
1	2249 (6.6)	1432 (6.7)	817 (7.7)	
Face AIS, *n* (%)				0.001
Injury not present	32,309 (94.2)	20,381 (94.9)	10,070 (94.6)	
1	1983 (5.8)	1086 (5.1)	575 (5.4)	
Neck AIS, *n* (%)				0.473
Injury not present	34,240 (99.8)	21,431 (99.8)	10,623 (99.8)	
1	52 (0.2)	36 (0.2)	22 (0.2)	
Spine AIS, *n* (%)				0.006
Injury not present	34,122 (99.5)	21,390 (99.6)	10,613 (99.7)	
1	170 (0.5)	77 (0.4)	32 (0.3)	
Thorax AIS, *n* (%)				< 0.001
Injury not present	32,965 (96.1)	20,890 (97.3)	10,391 (97.6)	
1	1327 (3.9)	577 (2.7)	254 (2.4)	
Abdomen AIS, *n* (%)				< 0.001
Injury not present	31,587 (92.1)	20,234 (94.3)	10,087 (94.8)	
1	1131 (3.3)	545 (2.5)	265 (2.5)	
2	1085 (3.2)	485 (2.3)	213 (2.0)	
3	354 (1.0)	153 (0.7)	67 (0.6)	
4	89 (0.3)	32 (0.1)	4 (0.0)	
5	4 (0.0)	1 (0.0)	0 (0.0)	
Missing	42 (0.1)	17 (0.1)	9 (0.1)	
Upper extremity AIS, *n* (%)				< 0.001
Injury not present	29,817 (87.0)	18,910 (88.1)	9306 (87.4)	
1	4475 (13.0)	2,557 (11.9)	1339 (12.6)	
Lower extremity AIS, *n* (%)				< 0.001
2	28,818 (84.0)	18,348 (85.5)	8891 (83.5)	
3	4590 (13.4)	2771 (12.9)	1582 (14.9)	
4	744 (2.2)	305 (1.4)	142 (1.3)	
5	135 (0.4)	42 (0.2)	28 (0.3)	
Missing	5 (0.0)	1 (0.0)	2 (0.0)	
External/other AIS, *n* (%)				< 0.001
Injury not present	33,429 (97.5)	21,068 (98.1)	10,458 (98.2)	
1	863 (2.5)	399 (1.9)	187 (1.8)	
Type of fracture, *n* (%)				
Ilium fracture	2472 (7.2)	1203 (5.6)	558 (5.2)	< 0.001
Ischium fracture	719 (2.1)	499 (2.3)	253 (2.4)	0.096
Pubis fracture	21,440 (62.5)	14,543 (67.7)	7196 (67.6)	< 0.001
Sacrum fracture	7992 (23.3)	4740 (22.1)	2206 (20.7)	< 0.001
Coccyx fracture	2655 (7.7)	1391 (6.5)	524 (4.9)	< 0.001
Acetabulum fracture	11,888 (34.7)	5717 (26.6)	2848 (26.8)	< 0.001
Other pelvic fracture	4306 (12.6)	2501 (11.7)	1331 (12.5)	0.004
Hypotension on admission, *n* (%)	562 (1.6)	310 (1.4)	175 (1.6)	0.177
Missing	894 (2.6)	631 (2.9)	320 (3.0)	
Tachycardia on admission, *n* (%)	4298 (12.5)	2670 (12.4)	1299 (12.2)	0.709
Missing	879 (2.6)	631 (2.9)	297 (2.8)	
Type of intervention, *n* (%)				
Fixation of fracture	4140 (12.1)	1182 (5.5)	400 (3.8)	< 0.001
Vascular surgery	127 (0.4)	50 (0.2)	15 (0.1)	< 0.001
Angiographic procedure	686 (2.0)	362 (1.7)	152 (1.4)	< 0.001
Laparotomy or pelvic packing for hemorrhage control	136 (0.4)	39 (0.2)	6 (0.1)	< 0.001

Non-frail, pre-frail, and frail are defined as OFS 0, OFS 1, and OFS ≥ 2. Hypotension is defined as a systolic blood pressure ≤ 90 mmHg. Tachycardia is defined as a pulse rate ≥ 100 beats per minute

*AIS* Abbreviated Injury Severity Score

Before adjusting for confounding variables, both pre-frail and frail patients had higher rates of in-hospital mortality (1.8% and 2.5% compared to 1.2%, *p* < 0.001), complications (4.2% and 4.5% compared to 3.9%, *p* = 0.015), and FTR (0.8% and 0.9% compared to 0.6%, *p* = 0.003), compared to non-frail patients (Table 3). After controlling for confounding variables, pre-frail patients exhibited a 56% higher risk of in-hospital mortality [adjusted IRR (95% CI): 1.56 (1.32–1.85), *p* < 0.001], a 20% higher risk of complications [adjusted IRR (95% CI): 1.20 (1.09–1.32), *p* < 0.001], a 44% greater risk of FTR [adjusted IRR (95% CI): 1.44 (1.12–1.84), *p* = 0.004], and an 8% increase in the risk of ICU admission [adjusted IRR (95% CI): 1.08 (1.02–1.14), *p* = 0.008], compared to non-frail patients. Furthermore, frailty was associated with an approximate doubling in the risk of in-hospital mortality [adjusted IRR (95% CI): 1.88 (1.54–2.30), *p* < 0.001], a 25% higher overall risk of complications [adjusted IRR (95% CI): 1.25 (1.10–1.42), *p* < 0.001], a 56% higher risk of FTR [adjusted IRR (95% CI): 1.56 (1.14–2.14), *p* = 0.006], and a 10% increased risk of ICU admission [adjusted IRR (95% CI): 1.10 (1.02–1.18), *p* = 0.014], compared to non-frail patients. The results did not differ significantly in any of the subgroup analyses (Table 4).

**Table 3:** Crude outcomes in geriatric patients with pelvic fractures

	Non-frail (*N* = 34,292)	Pre-frail (*N* = 21,467)	Frail (*N* = 10,645)	*p* value
In-hospital mortality, *n* (%)	414 (1.2)	397 (1.8)	269 (2.5)	< 0.001
Any complication, *n* (%)	1322 (3.9)	892 (4.2)	474 (4.5)	0.015
Myocardial infarction	100 (0.3)	77 (0.4)	34 (0.3)	0.391
Cardiac arrest with CPR	122 (0.4)	84 (0.4)	39 (0.4)	0.796
Stroke	55 (0.2)	36 (0.2)	21 (0.2)	0.720
Deep vein thrombosis	154 (0.4)	75 (0.3)	36 (0.3)	0.106
Pulmonary embolism	90 (0.3)	36 (0.2)	17 (0.2)	0.025
Acute respiratory distress syndrome	39 (0.1)	22 (0.1)	13 (0.1)	0.869
Urinary tract infection	307 (0.9)	219 (1.0)	94 (0.9)	0.275
Pneumonia	122 (0.4)	86 (0.4)	44 (0.4)	0.581
Surgical site infection	33 (0.1)	8 (0.0)	2 (0.0)	0.004
Sepsis	70 (0.2)	64 (0.3)	28 (0.3)	0.083
Decubitus ulcer	95 (0.3)	64 (0.3)	50 (0.5)	0.007
Unplanned intubation	149 (0.4)	99 (0.5)	62 (0.6)	0.146
Unplanned admission to the OR	62 (0.2)	19 (0.1)	8 (0.1)	0.003
Unplanned admission to the ICU	369 (1.1)	279 (1.3)	184 (1.7)	< 0.001
Failure-to-rescue, *n* (%)	215 (0.6)	172 (0.8)	97 (0.9)	0.003
ICU admission, *n* (%)	3907 (11.4)	2256 (10.5)	1106 (10.4)	< 0.001
Length of stay (days), median [IQR]	4.0 [3.0–6.0]	5.0 [3.0–6.0]	5.0 [3.0–7.0]	< 0.001
Missing, *n* (%)	331 (1.0)	185 (0.9)	70 (0.7)	

Non-frail, pre-frail, and frail are defined as OFS 0, OFS 1, and OFS ≥ 2

**Table 4:** Association between frailty and adverse outcomes in geriatric patients with pelvic fractures

Adverse outcome	Non-frail	Pre-frail IRR (95% CI)	*p* value	Frail IRR (95% CI)	*p* value
All patients (*N* = 66,404)					
In-hospital mortality	Reference	1.56 (1.32–1.85)	< 0.001	1.88 (1.54–2.30)	< 0.001
Any complication	Reference	1.20 (1.09–1.32)	< 0.001	1.25 (1.10–1.42)	< 0.001
Failure-to-rescue	Reference	1.44 (1.12–1.84)	0.004	1.56 (1.14–2.14)	0.006
ICU admission	Reference	1.08 (1.02–1.14)	0.008	1.10 (1.02–1.18)	0.014
Fractures caused by a GLF (*N* = 40,713)					
In-hospital mortality	Reference	1.61 (1.29–2.00)	< 0.001	1.97 (1.52–2.55)	< 0.001
Any complication	Reference	1.16 (1.02–1.32)	0.023	1.30 (1.10–1.53)	0.002
Failure-to-rescue	Reference	1.58 (1.13–2.19)	0.007	1.78 (1.19–2.69)	0.005
ICU admission	Reference	1.14 (1.06–1.24)	< 0.001	1.20 (1.09–1.33)	< 0.001
Conservative management (*N* = 59,653)					
In-hospital mortality	Reference	1.61 (1.34–1.93)	< 0.001	1.87 (1.50–2.33)	< 0.001
Any complication	Reference	1.27 (1.13–1.41)	< 0.001	1.31 (1.14–1.52)	< 0.001
Failure-to-rescue	Reference	1.57 (1.18–2.08)	0.002	1.70 (1.19–2.43)	0.004
ICU admission	Reference	1.10 (1.03–1.18)	0.006	1.09 (1.00–1.20)	0.049
Abdomen AIS ≤ 1 (*N* = 63,912)					
In-hospital mortality	Reference	1.59 (1.32–1.91)	< 0.001	1.98 (1.59–2.46)	< 0.001
Any complication	Reference	1.25 (1.13–1.38)	< 0.001	1.35 (1.18–1.54)	< 0.001
Failure-to-rescue	Reference	1.55 (1.18–2.03)	0.002	1.71 (1.22–2.42)	0.002
ICU admission	Reference	1.10 (1.03–1.17)	0.004	1.11 (1.03–1.21)	0.010

Non-frail, pre-frail, and frail are defined as OFS 0, OFS 1, and OFS ≥ 2

IRRs are calculated using Poisson regression models with robust standard errors. All analyses were adjusted for age, sex, race, highest AIS in each region, type of fracture, surgical and angiographic procedures, hypertension, previous myocardial infarction, history of peripheral vascular disease, cerebrovascular disease, dementia, chronic obstructive pulmonary disease, smoking status, chronic renal failure, diabetes mellitus, cirrhosis, coagulopathy, drug use disorder, alcohol use disorder, major psychiatric illness, and advanced directives limiting care. Missing data was managed using multiple imputation by chained equations

*IRR* incident rate ratio, *CI* Confidence Interval, *GLF* Ground-level fall, *AIS* Abbreviated injury severity score

## Discussion

Ground level falls constitute the most common mechanism of injury in the United States, and geriatric patients are most prone to sustaining injury via this mechanism [27]. Pelvic fractures, thus, constitute a relatively common injury pattern in geriatric patients [9, 28]. Consequently, a significant proportion of geriatric patients with pelvic fractures can be classified as frail, with 1 out of 6 patients being considered frail in the current investigation using a large national trauma database. The results of the Poisson regression analysis indicate that frail geriatric pelvic fracture patients suffer from an elevated risk of in-hospital mortality, complications, FTR, and ICU admission, highlighting the need for targeting care towards this more vulnerable patient population.

Pelvic fractures present a heterogeneous patient population with varying prognoses dependent on factors such as the type of fracture and preexisting comorbidities. The original OFS was developed for hip fracture patients; however, the current study suggests that this frailty score may also be applicable to the pelvic fracture population, given the similarities in age, burden of comorbidities, and level of frailty [7, 8, 13, 23]. The OFS itself is an objective and simple tool that makes use of five dichotomous variables that can be easily retrieved from past medical records or the patient without additional blood tests or intraoperative data. This reduces the risk of human error and intercoder variability.

Frailty refers to a clinical condition in which patients have a reduced physiological capacity to cope with external stressors due to the decline of several organ systems. This can lead to an increased risk of postoperative complications and mortality. Although frailty is typically associated with aging, it's worth noting that it is a distinct process and not a guaranteed consequence of getting older [15–18]. Frailty has also been proposed as a measure in orthopedic trauma to ascertain priority for advanced measures, such as preoperative optimization, multidisciplinary care, and post discharge rehabilitative therapy [29–31]. Previously, frailty has been found to be an independent risk factor for suffering fractures, especially pelvic fractures [20, 21]. Frail pelvic fracture patients have also been observed to exhibit an increased risk of mortality and non-home discharge as well as lower functional status at discharge [8, 19, 20].

As demonstrated by current and previous studies, frailty is relatively common among patients with pelvic fractures [8, 19]. Simultaneously, the incidence of pelvic fractures in geriatric patients is increasing, in large part due to rising life expectancy [4–6]. As the trend of population aging is not expected to diminish in either the short or long term [1–3], this cohort will increasingly strain healthcare systems around the world. To address this issue, risk stratification tools, such as the OFS, are needed to optimally allocate resources and minimize added cost. Such an approach enables early intervention by identifying patients with an elevated risk of decline.

Given the paucity of evidence for how to optimize care in this population [5, 14], there is little consensus on how to best manage pelvic fracture patients [8, 32]. Taking into account the similarity of pelvic fracture to hip fracture patients [5–13], it may be worth investigating interventions which have proven effective in the later cohort. Orthogeriatric care models in particular have previously been proposed as a potential option [33–35]. These models have been found to offer a cost-efficient method for reducing length of stay, mortality, and the risk of delirium among hip fracture patients in multiple systematic reviews [36–38].

Previous research conducted by Ramser et al. and Ravindrarajah et al., employing the electronic Frailty Index (eFI), has indicated an association between frailty and an elevated risk of both short- and long-term mortality among patients with pelvic fractures [8, 20]. Moreover, the findings of Perea et al. revealed that patients with pelvic fractures classified as frail according to the Clinical Frailty Scale (CFS), exhibited a lower functional status upon discharge and were more likely to be transferred to a skilled nursing facility [19]. Using the 6-Item Modified Frailty Index (MF-6), Pean et al. investigated the impact of frailty on patients with pelvic fractures, as well as other lower extremity fractures, undergoing surgical fixation. Their comprehensive analyses not only underscored the association of frailty with heightened mortality risk, major adverse events, readmission, and non-home discharge, but also with an extended length of stay [39].

Many tools have consequently been employed to measure frailty in patients who have suffered a pelvic fracture, such as the CFS, the eFI, and MF-6 [8, 19, 20, 39]; moreover, the Fried frailty phenotype (FP) has also been a common measure of frailty in orthopedic patients [40–42]. However, these indices have limitations that restrict their clinical utility. The, to an extent, subjective nature of the CFS, relying on a clinician’s judgment and observation of functional abilities and overall health [43], can lead to interobserver variation, especially among inexperienced users [44–47]. The need to evaluate functional ability and the limitations imposed by underlying comorbidities also pose challenges when used in emergency settings. The eFI requires a total 36 variables, while the risk score used by Ramser et al. required 35, including the comorbidities in the Elixhauser Comorbidity Index, which can make the calculation cumbersome and time consuming, leading to less usage in clinical practice [48, 49]. Additionally, the MF-6 requires blood test results to determine the presence of hypoalbuminemia [39]. The FP allows for a detailed characterization of frailty in patients [18], yet it can be difficult to assess factors such as slowness and physical activity in patients immobilized by pain or unintentional weight loss and exhaustion in a population where 15% of patients suffer from dementia. In contrast, the OFS stands out by only requiring five dichotomous variables. It can also be assessed immediately upon arrival in the emergency room, or prior to admission based on electronic medical records, without the need for further blood tests. This focus on simplicity also necessitates that some markers of frailty used in other indices, such as confusion, dementia, and the use of walking aids, have been excluded from the OFS; nevertheless, the inclusion of these variables did not result in a clinically significant improvement in the discriminatory ability of the OFS [23]. While treating physicians make individual clinical decisions, predictive tools can facilitate resource allocation, support objective decision-making, and enhance communication with patients and their families.

Nevertheless, this study has several limitations. Its retrospective nature limits the availability of certain variables such as long-term mortality, quality of life, and readmission. Additionally, it was not possible to adjust for perioperative and anesthesia-related confounders. There also remains a risk residual confounding, as well as non-differential misclassification due to reliance on ICD-10 codes for many of the variables. Despite these limitations, the study benefits from its extensive national sample size. The dataset provides a range of information that allows for adjustments to account for preadmission comorbidities and demographic and racial variations.

## Conclusion

Frail patients with pelvic fractures demonstrate a disproportionately increased risk of in-hospital mortality, complications, failure-to-rescue, and ICU admission. The OFS can be used to identify this cohort. Additional investigations are warranted to determine what measures are required to mitigate adverse events in this vulnerable patient population.

### Supplementary Information

Below is the link to the electronic supplementary material.

Supplementary file1 (DOCX 34 KB)
